# *Thelazia callipaeda* Ocular Infection

**DOI:** 10.4269/ajtmh.23-0330

**Published:** 2023-10-02

**Authors:** Chang Shen, Peifang Ren, Qing Xu, Hong Lu

**Affiliations:** ^1^ Department of Ophthalmology, The First Affiliated Hospital, Zhejiang University School of Medicine, Hangzhou, China;; ^2^ Department of Clinical Laboratory Parasitology, The First Affiliated Hospital, Zhejiang University School of Medicine, Hangzhou, China

A 26-year-old female from Hangzhou, Zhejiang Province, China, presented with a complaint of foreign body sensation in her left eye for 5 days. She did not own an animal and had no history of contact with one. She recalled only a camping trip about 2 months ago. Examination of the eye by slit-lamp biomicroscopy revealed slight hyperemia in the conjunctiva of her left eye, and several semihyaline nematodes wriggled in the conjunctiva sac (Figure 1, Supplemental Video 1). There was no obvious secretion, conjunctiva papilla, or follicle. Two nematodes were taken out by swab and then submitted to the infectious disease department laboratory for analysis and identification. In the following week, four additional nematodes were removed from the conjunctiva fornix of her left eye by two scrupulous ophthalmologists. The worms were identified as *Thelazia callipaeda*.^1^ Each worm had a typical scalariform buccal cavity and conical tail^2^ (Figure 2).

**Figure 1.**
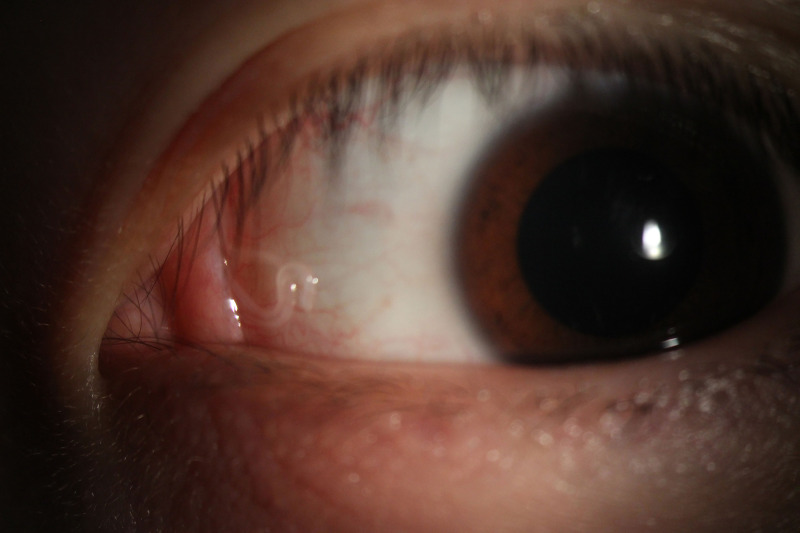
Anterior segment photography showed semihyaline nematodes wriggling in the conjunctival sac. There was slight hyperemia of the conjunctiva without other symptoms of the eye.

**Figure 2.**
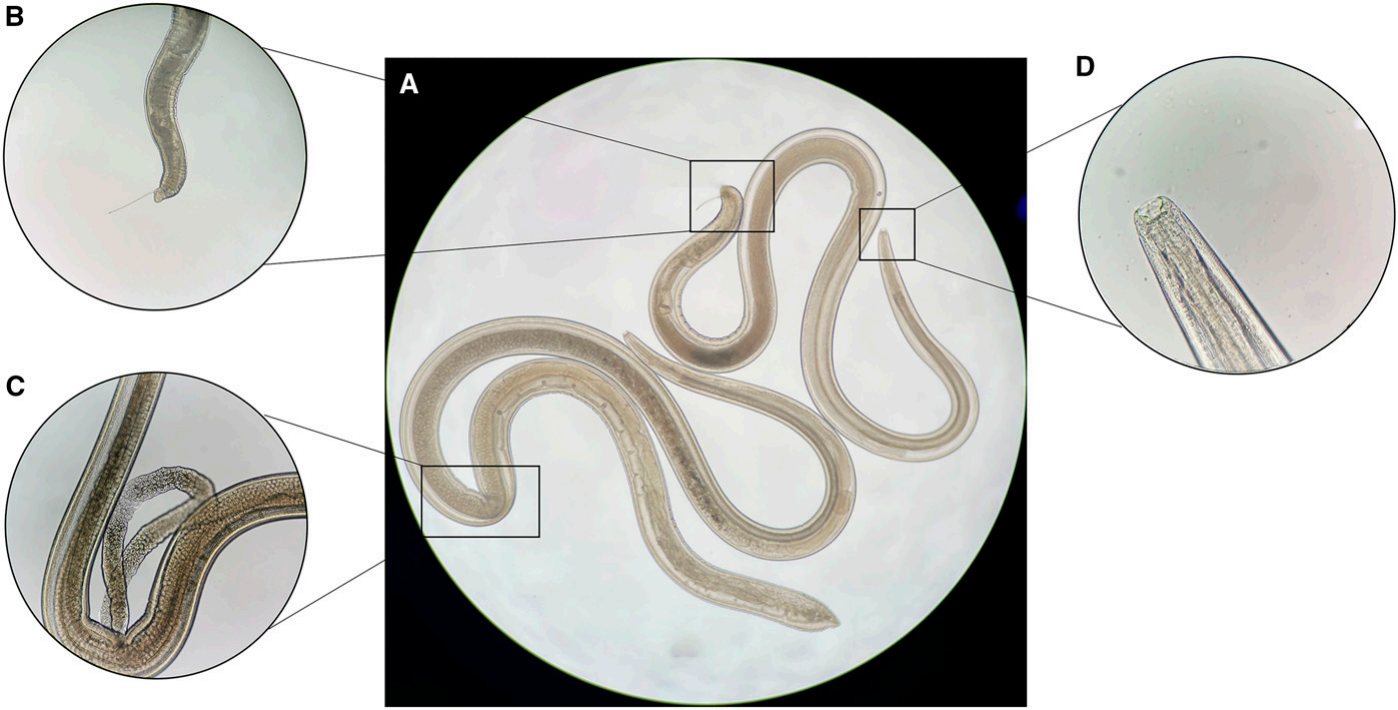
*Thelazia callipaeda* under microscope. (**A**) Two worms were removed from the patients’ conjunctival sac, and both worms had a conical tail as well as a scalariform buccal cavity, which is the key feature of *Thelazia callipaeda.* (**B**) The upper right worm was a male worm with a spiculum. (**C**) The lower left worm was a female with uterine tubules in the middle of the body, which contained several eggs. (**D**) The scalariform buccal cavity is shown.

## Supplemental files

10.4269/ajtmh.23-0330 Supplemental Materials
